# Mapping Current and Emerging Laboratory Techniques for Haemoglobinopathy Carrier Detection and Prevention: A Narrative Review from the HELIOS Action

**DOI:** 10.3390/ijms27093916

**Published:** 2026-04-28

**Authors:** Norafiza Mohd Yasin, Sotiroula Chatzimatthaiou, Adela Perolla, Adoracion Blanco Alvarez, Miguel Brito, Ghada El-Kamah, Merita Xhetani, Petros Kountouris, Coralea Stephanou, Jan Traeger-Synodinos

**Affiliations:** 1 Department of Clinical Genetics/LDGA, Leiden University Medical Center, 2300 RC Leiden, The Netherlands; 2 Haematology Unit, Cancer Research Centre (CaRC), Institute for Medical Research (IMR), National Institute for Health (NIH), Setia Alam 40170, Selangor, Malaysia; 3 Department of Blood Disorder Genetics and Thalassaemia, The Cyprus Institute of Neurology and Genetics, 1683 Nicosia, Cyprus; sotiroulac@cing.ac.cy (S.C.); petrosk@cing.ac.cy (P.K.); coraleas@cing.ac.cy (C.S.); 4 Faculty of Medicine, University of Medicine, 1005 Tirana, Albania; adelaperolla19@gmail.com; 5 Haematology Molecular Genetics Unit, Department of haematology, Hospital Universitari Vall d’Hebron (HUVH), Experimental Haematology, Vall d’Hebron Institute of Oncology (VHIO), 08035 Barcelona, Spain; adoracion.blanco@vallhebron.cat; 6 Health and Technology Research Center, Escola Superior de Saúde de Lisboa, Instituto Politécnico de Lisboa, 1990-096 Lisboa, Portugal; miguel.brito@estesl.ipl.pt; 7 Human Genetics and Genome Research Institute, National Research Centre, Cairo 12622, Egypt; ghadaelkamah@hotmail.com; 8 Department of Biology, Faculty of Natural Sciences, University of Tirana, 1009 Tirana, Albania; merita.xhetani@fshn.edu.al; 9 Center of Molecular Diagnostic and Genetics Research, University Hospital Obstetrics and Gynecology “Mbretëresha Geraldinë”, 1045 Tirana, Albania; 10 School of Medicine, National and Kapodistrian University of Athens, 11527 Athens, Greece; jtraeger@med.uoa.gr

**Keywords:** narrative review, thalassaemia, haemoglobinopathies, carrier screening, laboratory diagnostics, global epidemiology, prevention strategies

## Abstract

Haemoglobinopathies remain a major public health challenge, predominantly in endemic regions. Increasing migration has extended their prevalence in previously non-endemic areas, complicating early detection and prevention. As part of the HELIOS CA22119 COST Action Working Group 1, this narrative review critically examines established and emerging laboratory techniques for haemoglobinopathy carrier detection. It also explores diagnostic limitations, regional disparities, and opportunities for global harmonisation to support early detection, prevention, and equitable care. A literature search of MEDLINE, PubMed, Scopus, and EMBASE (2014–2024) identified studies on the screening, diagnosis, and prevention of haemoglobinopathies. Findings were synthesised across three domains: (1) preventive strategies, (2) carrier screening methods, and (3) prenatal diagnostic approaches. Advances in molecular technologies have improved diagnostic sensitivity and specificity. However, conventional haematological approaches, particularly complete blood count and haemoglobin typing, remain essential and cost-effective first-line tools. Key challenges include unequal access to advanced diagnostics and the lack of standardised protocols across regions. Strengthening prevention requires coordinated global efforts to promote accessible, accurate, and standardised diagnostic approaches tailored to regional genomic, economic, and healthcare contexts. Early and equitable carrier detection, combined with effective prenatal diagnosis, is critical to reducing the global burden and improving health outcomes in both endemic and emerging regions.

## 1. Introduction

Haemoglobinopathies, including thalassaemia and sickle cell disease (SCD), are among the most prevalent monogenic disorders worldwide, particularly in malaria-endemic regions such as the Mediterranean, Middle East, Africa, and South Asia, reflecting historical selective advantages. Each year, approximately 300,000–400,000 infants are born with severe haemoglobin disorders [1], including more than 40,000 affected by β-thalassaemia, of whom around 25,500 are transfusion-dependent [2]. These disorders result from pathogenic variants affecting the α- and β-globin gene clusters. To date, over 3000 globin gene variants have been identified, encompassing both disease-causing mutations and common polymorphism [3,4].

**Figure 1:**
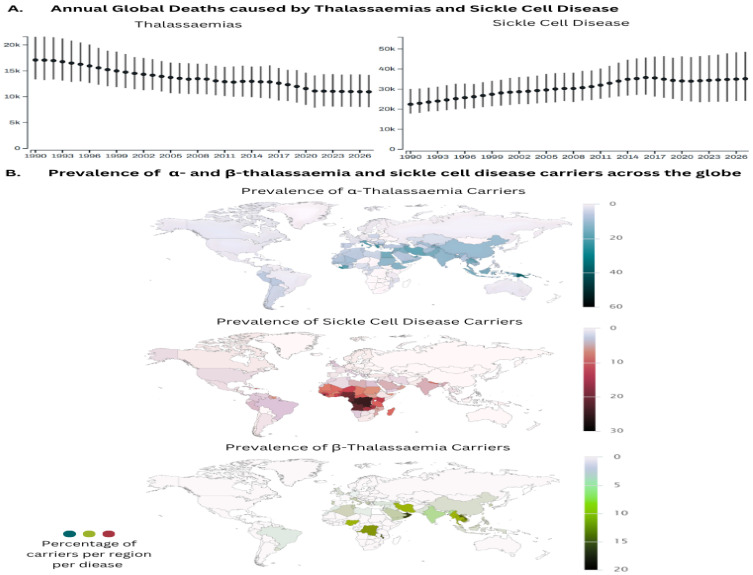
Global prevalence of thalassaemias and sickle cell carriers, and deaths caused by the disorders. (**A**) The graphs present the annual deaths (both sexes, all ages) recorded caused by thalassaemias and sickle cell disorders according to the GBD (Source: Institute for Health Metrics and Evaluation. Used with permission. All rights reserved). (**B**) Maps show the distribution of α- and β-thalassaemia carriers (both sexes, all ages) across the globe according to data gathered by IthaMaps [8] (accessed September 2025).

Although mortality from thalassaemia and SCD has plateaued in recent years, absolute numbers remain high (Figure 1A) [5]. Migration has expanded their distribution to non-endemic regions (Figure 1B, Supplementary Table S1) such as Northern Europe, North America, and Australia [6,7], underscoring the need for effective public health strategies, including carrier detection, identification of at-risk couples, prenatal diagnosis, and management for affected pregnancies. Their molecular and phenotypic heterogeneity, involving diverse α- and β-globin gene variants (Supplementary Figures S1 and S2, Table S2), further complicates diagnosis and highlights the importance of accurate and accessible screening.

Carrier identification is a cornerstone of preventive strategies, enabling informed reproductive choices, genetic counselling, prenatal diagnosis, and timely intervention. Traditional methods such as complete blood counts (CBC) and haemoglobin typing by capillary electrophoresis (CE), or high-performance liquid chromatography (HPLC), and targeted molecular approaches, including Amplification Refractory Mutation System Polymerase Chain Reaction (ARMS-PCR), Gap-PCR, reverse dot blot (RDB), and high-resolution melting (HRM) analysis, efficiently detect common variants and are widely used in routine screening. For more comprehensive analysis, Sanger sequencing and multiplex ligation-dependent probe amplification (MLPA) are employed [9]. However, targeted methods may miss uncommon variants. Moreover, Sanger sequencing is labour-intensive, and MLPA cannot fully resolve complex structural changes [9,10]. Emerging technologies, including next-generation sequencing (NGS) and third-generation long-read platforms (e.g., Oxford Nanopore, PacBio), enable high-throughput, comprehensive analysis of single nucleotide variants (SNVs) and structural variants (SVs), with the latter including copy number variations (CNVs), offering improved sensitivity, specificity, and scalability, particularly in genetically diverse populations [10].

Despite technological advances, the global harmonisation of screening and diagnosis remains limited by disparities in accessibility, laboratory infrastructure, cost, and population-specific variants. This review summarises established and emerging laboratory techniques, highlights limitations and regional disparities, and discusses strategies to standardise carrier detection and prenatal diagnosis, ultimately supporting early detection, prevention, and equitable care worldwide.

## 2. Review Methodology

### 2.1. Defining the Scope and Research Question

As part of the HELIOS CA22119 COST Action [11] (HELIOS Action: advancing research, education & equity in haemoglobinopathies; in press, *HemaSphere*) Working Group 1 (WG1), this review maps current and emerging laboratory techniques for haemoglobinopathy screening, diagnosis, and prevention. This collaboration specifically focused on establishing an excellent network to improve care, research and equity for patients with sickle cell disease and thalassaemia across Europe and beyond. The study scope was refined around four key thematic areas: preventive strategies, haematology diagnostic methods, molecular diagnostic methods and prenatal diagnosis. Using elements of the PICO framework, the review considers individuals affected by haemoglobinopathies (*Population*) and explores laboratory-based screening and diagnostic methods (*Intervention*), and the identification of current practices and the potential for the harmonisation of practices (*Outcome*).

### 2.2. Literature Search Strategy

A comprehensive literature search was conducted in November 2024 across MEDLINE, Scopus, PubMed, and EMBASE, covering publications from 2014 to 2024. The strategy, developed by consensus, used free-text terms such as *thalassaemia*, *haemoglobinopathy*, *sickle cell*, *screening*, *diagnosis*, *laboratory techniques*, *guidelines*, *and prevention* (Supplementary Table S3). Where relevant literature was limited, key studies published before 2014 were included. Targeted supplementary searches addressed gaps, including prevention programmes in underrepresented countries and region-specific emerging diagnostic techniques, guided by both dataset needs and the authors’ expertise.

### 2.3. Inclusion and Exclusion Criteria

Articles were included if they addressed: (a) screening (haematological, biochemical, or molecular methods); (b) emerging approaches, including next- and third-generation sequencing; (c) epidemiological data relevant to screening practices (genotypes, allele frequencies); (d) prevention strategies (e.g., premarital, preconception, school-based, or targeted approaches); (e) prenatal diagnosis; and (f) expanded carrier screening. Exclusion criteria were case reports, abstracts without full-text, unpublished or non-peer-reviewed studies, and papers focused primarily on newborn screening (NBS) or preimplantation genetic testing. Only English-language studies were considered. Grey literature, including policy reports, was consulted to capture recent data on prevention strategies.

### 2.4. Study Selection Process

All references were imported into Rayyan [12], with duplicates removed. Titles and abstracts were independently screened in pairs by eight reviewers (FMY, SC, AP, ABA, MB, GEK, MX, and CS) against the predefined thematic areas, and non-relevant records were excluded. Full texts of potentially eligible articles were then assessed by the same reviewers within their assigned thematic areas. Discrepancies were resolved by group consensus.

### 2.5. Data Extraction and Synthesis

Data were collaboratively extracted using a structured spreadsheet (Google LLC, Mountain View, CA, USA) and reviewed as a group. Publications were categorised by country using World Bank income classifications and regional groupings reflecting disease burden, diagnostic capacity, and health policy, guiding the analysis of screening strategies, implementation challenges, and diagnostic technology adoption. Geographic data were visualised with mapchart.net (MapChart GmbH, Dresden, Germany).

## 3. Thematic Overview of the Evidence

### 3.1. Study Characteristics

**Figure 2:**
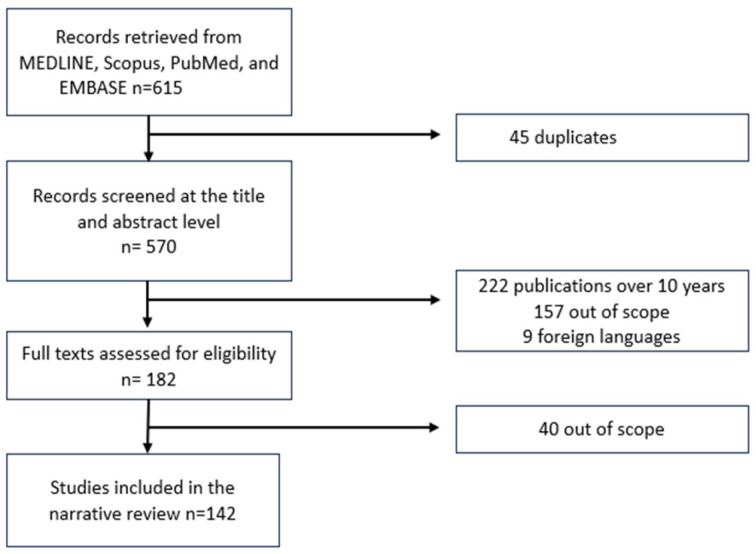
Flow diagram of the narrative review: the publication review step process.

After duplicate removal, 582 abstracts were screened, with 399 excluded as out of scope. For the remaining 183 articles, the full texts were assessed, with 41 excluded as out of scope. Ultimately, 142 articles were included in the narrative review (Figure 2).

### 3.2. Prevention Policies

**Figure 3:**
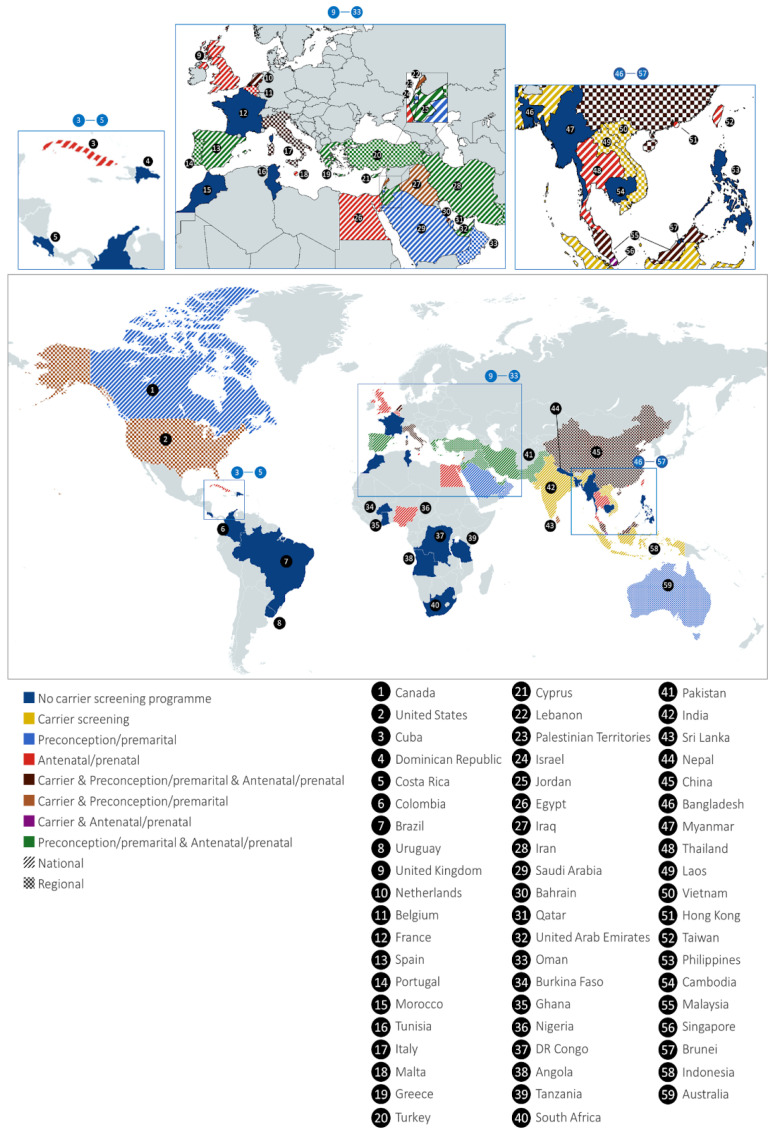
Global distribution of carrier screening programmes for haemoglobinopathies: the figure summarises the status and application of different prevention strategies across the globe based on the publication reviewed by this narrative review.

In high-burden regions, national or regional carrier screening programmes have demonstrated measurable success, particularly when focused on premarital and preconception screening rather than antenatal detection (Figure 3). Cyprus provides a longstanding example: since the 1970s, a comprehensive programme combining public education, mandatory premarital screening, genetic counselling, and prenatal diagnosis reduced homozygous β-thalassaemia births within a decade [13,14,15]. Similar outcomes were reported in Italy (Sardinia) and Greece, where premarital and school-based screening led to measurable declines in transfusion-dependent thalassaemia (TDT) incidence [15,16]. These efforts were justified on both public health and economic grounds, as prevention proved more cost-effective than lifelong transfusion and chelation.

The review of active prevention programmes shows notable differences across regions and income levels (Supplementary Table S4). Structured carrier screening, premarital, preconception, or antenatal/prenatal, is reported more frequently in high-income countries (HICs) (52.5%, 21/40) than in upper-middle (UMICs) (22.5%, 9/40) or lower-middle-income (25%, 10/40) countries (LMICs). Twelve countries, mostly high- and upper-middle-income (e.g., Cyprus, Iran, Saudi Arabia), have mandatory screening, while others, including Italy (Sardinia), Greece, Malaysia, and the United Kingdom, offer voluntary programmes. By contrast, half of low- and lower-middle-income countries (10/20), including Angola, Bangladesh, DR Congo, Nepal, Philippines, and Tanzania, lack carrier screening despite high disease burden. In countries like India, Jordan, and Nigeria, screening initiatives exist but are limited, targeted, or inconsistently implemented, and only a few low-income countries report pilot or regional initiatives.

### 3.3. Laboratory Haematology Standards (Cut-Off Values, Hb Typing, HbA2 Quantification)

Haemoglobinopathies are unique among genetic disorders because carrier detection can be achieved through haematological and biochemical tests, rather than relying solely on DNA analysis [9]. Standard screening combines CBC with haemoglobin concentration (Hb), mean corpuscular volume (MCV, fL), mean corpuscular haemoglobin (MCH, pg), and red blood cell (RBC) count (×10^12^/L), with HbA_2_ quantification for β-thalassaemia detection. Cutoffs are crucial for screening accuracy, typically set at two standard deviations (SDs) below the mean MCV and MCH of the normal population, with most centres using MCV < 80 fL and/or MCH < 27 pg (Table 1a). Excluding iron-deficiency anaemia is essential, particularly in high-prevalence regions [7,10]. Some programmes, such as in Guangdong and Southern Jiangxi, China, use higher cutoffs (MCV < 82 fL, MCH < 27 pg) based on local data, followed by genetic testing, which increases sensitivity but reduces specificity [17].

Guidelines generally recommend MCV 78-79 fL and MCH 27 pg (Table 1b), but optimal thresholds should be determined locally, considering disease prevalence, mutation spectrum, and programme objectives. MCH is more stable than MCV, since MCV is affected by sample storage; CBC analysis should ideally be performed within six hours. Using stricter cutoffs (MCV < 78 fL, MCH < 25 pg) can reduce false positives and improve resource use, though low MCV is less effective for detecting Hb E (HBB:c.79G>A) and Hb S (HBB:c.20A>T) carriers, single α-globin gene deletions −α^3.7^ (NG_000006.1:g.34247_38050del) and −α^4.2^ (NC_000016.10:g.169818_174075del), or most non-deletional α-globin gene mutations [18,19]. Importantly, the performance of haematological indices differs between α- and β-thalassaemia carriers. In β-thalassaemia traits, reductions in MCV and MCH are typically more pronounced, making these indices reliable first-line screening tools. In contrast, individuals with single α-globin gene deletions (silent carriers) may present with borderline or even normal red cell indices, significantly reducing detection sensitivity. As a result, reliance solely on haematological cut-offs may lead to underdiagnosis of α-thalassaemia carriers, particularly in screening programmes that do not incorporate molecular testing. Secondary cutoffs derived from receiver operating characteristic (ROC) curve analysis can further distinguish α^+^-thalassaemia from normal controls, and α^0^- versus α^+^-thalassaemia heterozygotes (Table 1a), although these approaches do not fully overcome the limitations of red cell indices.

**Table 1:** Comparison of cut-off values reported in studies and guidelines.

Table 1a: The cut-off value reported in several studies for carrier identification						
Country/ Region	Cut-off Values	Sensitivity	Specificity	Study Population	Diagnosis	Reference(s)
**Lower-Middle-Income Countries**						
India [New Delhi]	MCV ≤ 72 fL [low cut-off]	63.2%	68.3%	1300 pregnant women [antenatal study]	β-thalassaemia	[20]
	MCV < 80 fL [high cutoff]	78.8%	44.3%			
	MCH ≤ 24 pg [low cut-off]	63.6%	59.4%			
	MCH < 27 pg [high cut-off]	77.3%	36.1%			
	Combined cut-off MCV < 80 fL and MCH < 27 pg	89.4%	16.4%			
	Combined cut-off MCV < 74 fL and MCH < 28 pg	94.0%	21.2%			
Indonesia (Sumatra)	MCH < 20.5 pg [cut-off for β^0^-thal]	85%	90%	203 subjects with heterozygous β-thalassaemia [β^0^ and β^+^]	β-thalassaemia	[21]
	MCV < 66.8 fL [cut-off for β^0^-thal]	87%	87%			
	HbA_2_ > 4.65% [cut-off for β^0^-thal]	88%	74%			
Egypt (Cairo)	HbA_2_ > 3.5%	100%	70%	280 microcytic hypochromic cases	β-thalassaemia	[22]
	HbA_2_ > 4%	97.4%	72.7%			
**Upper-Middle-Income Countries**						
Thailand	MCV < 76.15 fL [secondary cut-off]	100%	60.9%	438 pregnant women with MCV < 80 fL	α^0^-thalassaemia	[23]
	MCH < 26.5 pg	95.2%	82.3%	396 pregnant women	α^0^- and β-thalassaemia	[24]
	HbA_2_ ≥ 4.0%	100%	99.6%	418	β-thalassaemia	[25]
China	Positive haematology and HbA2	80.64%	90.74%	1093 Han males & females of childbearing age	α- and β-thalassaemia	[26]
	MCV and MCH	69.38%	94.36%			
	HbA_2_	43.58%	94.37%			
	MCV < 80 fL and MCH < 27 pg HbA_2_ > 3.5%	Not studied		11,043 pre-pregnancy screening	Positive haematology β-thalassaemia	[27]
	MCV < 82 fL or MCH < 27 HbA_2_ > 3.5% HbA_2_ < 2.5% or HbF < 2%	Not studied		10,285 from the outpatient department/136,312 residents of reproductive age	α- and β-thalassaemia	[18,28,29]
Lebanon	CBC, sickling test [1st line] Capillary electrophoresis [CE] [2nd line]	Not studied		184,105 subjects screened during the 12-year period	Sickle cell	[30]
Malaysia (National)	MCH ≤ 27 pg [initial screening adopted based on BCSH guideline 2010]			622 confirmed as heterozygous α^0^- or α^+^-thalassaemia	α^0^-thalassaemia	[31]
	MCH ≤ 23.5 pg [prediction of α^0^ thalassaemia]	98%	85%			
	MCH ≥ 25 pg [prediction of α^0^ thalassaemia]	98%	50%			
Turkey (Samsun)	MCV ≤ 80 fL, MCH ≤ 27 pg, and HbA2 ≥ 3.5% [β-thal] HbA_2_: 3.1–3.4% [borderline β-thal]	Not studied		52,338 individuals screened [premarital]	β-thalassaemia	[20]
Turkey (Nigde)	HbA_2_: 3.3–3.8% [borderline] With MCV ≤ 80 fL MCH ≤ 27 pg	Not studied		2013 individuals screened [premarital]	β-thalassaemia	[32]
**High-Income Countries**						
Italy	MCV ≤ 82 fL MCH ≤ 27 pg HbA_2_ > 3.2% HbA_2_ < 2%	Not studied		NA	α- and β-thalassaemia	[33]
	MCV < 80/82 fL and or MCH < 27 pg Low or normal HbA_2_ 2.0–3.1% HbA_2_ > 3.5%	Not studied		2649 subjects	α- and β-thalassaemia	[11]
Netherland	MCV < 80 fL MCH < 27 pg HbA_2_ > 3.5%	Not studied		NA	α- and β-thalassaemia	[1]
Spain	MCV 82.05 fL	94.7%	94.9% [AUC:0.975]	174 alpha thalassaemia	α^+^-thalassaemia vs. normal control α^0^-thalassaemia vs. α^+^-thalassaemia	[34]
	MCH 27.35 pg	94.7%	92.3% [AUC:0.982]			
	MCV 74.0 fL	85.70%	78.50% [AUC:0.879]			
	MCH 23.40 pg	85.70	78.50% [AUC:0.905]			
Singapore	MCH ≤ 28 pg	100%	15.38%	10,084 non-antenatal and 11,364 antenatal samples	α- and β-thalassaemia	[35]
	MCV ≤ 80 fL	97.62%	15.38%			
United Kingdom	MCH ≤ 27 pg and Family Original Questionnaire (FOQ) (country with high prevalence) MCH < 25 pg + FOC (country with high prevalence) HbA2 ≥ 3.5%	Not studied		A review on SCD management in pregnancy	α- and β-thalassaemia α^0^-thalassaemia β-thalassaemia	[36]
**Table 1b: The cut-off value reported in several guidelines**						
**Guidelines**	**Cut-off Values**	**Recommendation**		**Study population**	**Diagnosis**	**Reference (s)**
EMQN Best Practice Guidelines	Reduced red cell indices (MCV < 79 fL, MCH < 27 pg)	Best practice		Suggest establishing own ranges	Carrier screening	[10]
	HbA_2_ > 3.5% is the standard cut-off value				β-thalassaemia	
	HbA_2_ of 3.1–3.5%			Depends upon the method, reference range, and CV	Borderline β-thalassaemia	
Thalassaemia International Federation Guidelines 2025	MCV < 80 fL and/or MCH < 27 pg with normal Ferritin	NA			Thalassaemia carrier (α and β)	[37]
	HbA_2_ ≥ 4% and HbF: 1–5%				β-thalassaemia	
Clinical Practice Guidelines for Management of Thalassaemia (2nd Edition)-Malaysian CPG	MCV ≤ 80 fL and/or MCH ≤ 27 pg with normal Hb level	NA		School screening programme—National level	Thalassaemia carrier	[31]
	HbA_2_ ≥ 4% HbA_2_: 3.3–3.9%				β-thalassaemia. Borderline β-thal	
A British Society for Haematology Guideline	MCH < 27 pg, HbA_2_ ≥ 3.5% >4%, Normal MCV	High endemic		Antenatal screening/pregnant women	β-thalassaemia, Mild β-thalassaemia	[36]
	MCH < 27 pg ± Hb typing	Low endemic		If the biological father’s origin is from an endemic or unknown population	Suspected thalassaemia	
	MCH < 27 pg	Antenatal setting		Regardless of iron status (insufficient time in the antenatal setting)	Suspected α-thalassaemia	

The percentage of HbA_2_ remains the most valuable marker for β-thalassaemia carriers. Although several methods exist, only a few are recommended for accurate measurement. Reported HbA_2_ cutoffs range from 3.1% to 3.5% (Table 1), with most centres using >3.5% to indicate heterozygous β-thalassaemia, yielding 100% sensitivity and 70% specificity; raising the threshold to 4.0% may reduce sensitivity but improve specificity [38]. Borderline HbA_2_ elevation can result from acquired conditions, such as antiretroviral therapy, hyperthyroidism, megaloblastic anaemia, or co-eluting haemoglobin variants, while silent or mild β-variants can also slightly increase HbA_2_ levels [7]. At the molecular level, borderline HbA2 levels are often associated with mild or ‘silent’ β^+-^ thalassaemia mutations such as promoter (i.e., Cap + 1, HBB:c.-50A>C) or polyadenylation signal variants (HBB:c.*+110T>C, c.*+111A>G, c.*+112A>G, c.*+113A>G) that only partially reduce β-globin synthesis, resulting in a modest compensatory increase in δ-globin chain production and hence HbA2. In addition, co-inheritance of α- and δ-thalassaemia may reduce the relative imbalance between α- and non-α globin chains, thereby attenuating HbA2 elevation. These interacting genetic factors can lead to HbA2 values within the borderline range (approximately 3.1–3.9%), complicating carrier detection and necessitating confirmatory analysis [9].

Most laboratories use CE and/or HPLC for haemoglobin analysis, with confirmation recommended using two complementary methods for accurate variant identification. However, with advances in molecular diagnostics, many centres increasingly rely on molecular testing for definitive diagnosis, particularly in cases with inconclusive haematological findings or suspected α-thalassaemia.

### 3.4. Indications for Molecular Genotyping

Following haematology testing, definitive genotyping of likely or suspected heterozygotes is essential for genetic counselling, risk assessment, and prenatal diagnosis, yet it is not universally implemented. In many centres, particularly those with limited resources, DNA analysis is reserved for selected cases rather than integrated into routine screening. When haematological indices and haemoglobin electrophoresis (e.g., HbA_2_ > 4%) clearly indicate a classical β-thalassaemia trait, molecular testing may be considered unnecessary [7,10]. However, genotyping is crucial when results are inconclusive or borderline (e.g., HbA_2_ 3.1–3.9%), when both partners are carriers and foetal risk must be assessed, or when complex genotypes or rare mutations are suspected that are not detectable by standard techniques [10,11].

The diagnosis of α-thalassaemia heterozygosity almost always requires DNA-based testing, as CBC and haemoglobin analysis alone are insufficient [10,11]. Silent carriers or individuals with compound α-globin gene deletions may present with only mild hypochromic microcytosis, risking missed or incorrect diagnosis without molecular testing. Definitive genotyping ensures accurate reproductive risk assessment and enables targeted prenatal diagnosis.

While molecular testing may not be required for all individuals, it remains a cornerstone in managing reproductive choices for at-risk couples and preventing severe or transfusion-dependent haemoglobinopathies. Differentiating between cis (−−/αα) and trans (−α/−α) forms of alpha thalassaemia is essential for accurate genetic counselling, particularly in assessing the risks of hydrops foetalis and Hb H disease. These conditions can only be definitively diagnosed through molecular characterisation. Centres with advanced resources may include genotyping in routine screening, whereas others adopt a tiered approach, using it as a second-line test based on haematological findings. Advances in molecular technologies now permit reliable detection of diverse α- and β-globin gene variants, although testing strategies continue to vary across centres depending on local mutation prevalence, resources, and laboratory capacity.

### 3.5. Current Molecular Genotyping Techniques for Haemoglobinopathies

Molecular diagnosis is fundamental for prevention, particularly in regions with high carrier frequencies. This section focuses on methods for detecting SVs (including CNVs) and SNVs, emphasising performance, limitations, and adaptability to local contexts.

Most DNA-based testing relies on PCR. In resource-limited settings, standard targeted methods, including Gap-PCR, ARMS-PCR, and RDB, can be optimised for common ethnic-specific mutations and are cost-effective and are technically straightforward to apply [1,7]. Methods that screen specific gene regions, such as HRM, can flag sequence variations but require confirmation by targeted methods or Sanger sequencing. Ethnic background and family history (including consanguinity) support diagnostic strategies.

The choice between targeted panels, broader gene screening, and comprehensive sequencing depends on population genetic diversity, existing screening programmes, infrastructure, and cost. Commonly applied techniques are summarised below.

#### 3.5.1. Targeted PCR-Based Methods

Gap-PCR detects large deletions in the α- and β-globin loci [18,39,40] using primer pairs that flank established breakpoints. This technique produces PCR products with very specific sizes that can be viewed using conventional agarose gel electrophoresis. It is rapid, inexpensive, reproducible, and readily multiplexed for population-specific variants [27,41], making it well-suited for mass screening where deletion spectra are well-characterised [42]. ARMS-PCR (Amplification Refractory Mutation System PCR) is a seminal method for locating known single-nucleotide variants (SNVs), first applied for beta-globin variants in 1990. It has been demonstrated to be a reliable and extremely cost-effective method, especially for identifying beta globin variants [6,43,44,45,46]. RDB combines multiplex PCR with the membrane-based hybridisation of oligonucleotide probes designed to be complementary to the variants under investigation, with the potential to detect up to ~20 predetermined *HBA* and *HBB* variants simultaneously [47]. It offers high throughput with good specificity and sensitivity but requires specialised membranes, labelled probes, and stringent post-PCR hybridisation conditions, making it more suitable for reference laboratories or national screening hubs [39,48]. HRM-based assays can detect both SVs and SNVs according to the design and even in a multiplex format in one closed-tube reaction. Each mutation produces a unique melting curve with distinct melting temperatures (Tm), enabling the simultaneous detection of alpha and beta globin genes with 100% concordance to multiplex real-time PCR results [32].

#### 3.5.2. Generic Methods

Sanger sequencing still remains the gold standard for identifying or verifying globin genotypes, especially uncommon or unique variants or complex genotypes, or when haematologic indices are inconclusive. However, it tends to be more time-consuming, technically demanding and costly per test [1,6,48]. On the other hand, MLPA has been demonstrated as a robust method used for detecting known or unknown CNVs involving α-, β-, and δβ-thalassaemia variants. The technology is based on the hybridization of multiple probe-pairs across the entire locus of interest, followed by ligation of those pairs that find a complimentary sequence in the sample, and finally amplification of the ligated probe-pairs. The latter is facilitated by universal-tag PCR primers for all probe pairs and subsequently the analysis of fragment sizes. The technical strength of MLPA makes it highly suited for detecting heterozygous or homozygous deletions or duplications. It offers high multiplexing by analysing multiple targets in one tube [49,50]. Importantly, the choice of diagnostic methodology differs between α- and β-thalassaemia due to their underlying mutation spectra. α-thalassaemia is predominantly caused by structural variants (SVs), particularly gene deletions involving *HBA1* and *HBA2* loci, whereas β-thalassaemia and other haemoglobinopathies more commonly associated with single nucleotide variants (SNVs) or small insertions/deletions. As such, methods capable of detecting copy number variation, such as MLPA, are particularly critical in the diagnostic workflow for α-thalassaemia, while sequencing-based approaches are often prioritised for β-globin gene analysis. This distinction underpins the selection of appropriate molecular strategies in routine diagnostics. An overview of thalassaemia diagnostic methodologies across countries and a comparison of molecular screening methodologies for haemoglobinopathies are presented in Table 2 and Table 3 respectively.

### 3.6. Emerging Molecular Genotyping Methods for Haemoglobinopathies

Current diagnostic methods for thalassaemia and haemoglobin variants have advanced significantly, covering the vast majority of clinically relevant variants. Emerging molecular techniques promise further improvements in efficiency and throughput, potentially transforming laboratory workflows.

The most notable innovation is NGS. NGS (or Massively Parallel Sequencing) delivers faster turnaround times and high sensitivity (~99.9%) and specificity compared to traditional methods [65], particularly for complex thalassaemia genotypes. It is especially well-suited for detecting SNVs and small indels, which are the predominant mutation types in β-thalassaemia and many haemoglobin variants. However, short-read NGS struggles with accurately identifying SVs, including gene rearrangements, large deletions, and duplications, as well as highly repetitive regions, such as the Alu and SINE repeats in the *HBA1* and *HBA2* gene clusters, which are particularly relevant in α-thalassaemia. Consequently, while NGS may serve as a comprehensive first-line tool for β-globin gene analysis, its ability to detect structural variants remains limited, and additional methods may still be required to fully resolve α-globin gene alterations. Commercial NGS kits, like the Devyser Thalassaemia NGS Assay (Devyser, Hagersten, Sweden), are increasingly adopted in centres, primarily in high- and upper-middle-income countries [65,66]. As costs decline, NGS represents a streamlined and comprehensive alternative to conventional stepwise genotyping, although as all emerging technologies, it will require monitoring and further validation.

Third-Generation Sequencing (TGS), also known as Long-Read Sequencing (LRS) addresses the limitations of short-read NGS. TGS sequences long DNA molecules without amplification or fragmentation, allowing simultaneous and accurate detection of SNVs, and simple and complex SVs [67], addressing even the usual limitations posed by highly homologous regions such as the *HBA2* and *HBA1* genes [68]. Targeted LRS improves detection for rare or previously unreported and/or complex SVs [65], and the first reports indicate that it is highly effective for carrier screening [69,70], enabling the comprehensive identification of SNVs and SVs underlying haemoglobinopthies. One study demonstrated the utility of LRS screening at-risk couples with complex genotypes involving SNVs and SVs of both α- and ꞵ-globin genes to minimise the risk of misdiagnosis [66]. A specifically developed LRS protocol known as the Comprehensive Analysis of Thalassaemia Alleles (CATSA) combines LRS with custom primers and bioinformatic analysis to detect a wide range of thalassaemia-related genetic variants, including single nucleotide variants, indels, and large deletions, which are often missed by conventional methods. A comparison of emerging laboratory technologies is provided in Table 4. In summary, LRS protocols represent powerful diagnostic tools, particularly in complex or inconclusive cases or in research settings requiring comprehensive genotype resolution.

### 3.7. Prenatal Diagnosis (PND)

PND refers to the detection of foetal genetic alterations in high-risk couples, following prior identification of carrier status and provision of appropriate counselling in at-risk couples. It involves the analysis of foetal material obtained through invasive procedures such as chorionic villus sampling (CVS, 10–13 weeks), amniocentesis (15–20 weeks), or, rarely, cord blood collection (>18 weeks). Noninvasive assessment of cell-free foetal DNA in maternal circulation is being explored for screening on-going pregnancies, although current evidence does not yet support its routine diagnostic use.

The choice of sampling method depends on the technical and human resource capacity of each site, always in collaboration with obstetrics and gynaecology services. All PND procedures require comprehensive counselling on potential risks, diagnostic accuracy, implications of positive results, and reproductive options, including pregnancy termination where legally and socially permitted.

PND primarily targets clinically relevant haemoglobinopathies such as SCD and severe α- or β-thalassaemia. Although Hb Bart’s hydrops fetalis (caused by genotypes leading to severe or complete α-globin chain deficiency) often results in perinatal death, prenatal diagnosis remains valuable to support the management of associated maternal risks such as toxaemia and postpartum haemorrhage [52]. In contrast, PND for SCD is complicated by its variable clinical presentation [79], which limits prediction of disease severity and hinders informed parental decision-making. Identification of genetic modifiers (e.g., elevated HbF levels or co-inheritance of α-thalassaemia) may in future enhance prognostic accuracy [80].

PND and pregnancy termination for haemoglobinopathies are implemented in many countries through national or regional programmes. However, their structure and acceptance vary widely, and according to healthcare resources, legislation, religion, and cultural norms. Early programmes began in Greece (1974) [81], Cyprus, and Sardinia, expanding in subsequent decades across the Mediterranean, Middle East, and Asia [82,83] and later to other parts of Europe and Africa [84].

Foetal DNA analysis techniques are the same as those applied for genotyping carriers (and patients), selected and performed according to the laboratory capacities of each site. The main molecular methods for genotyping the foetal samples for prenatal diagnosis of haemoglobinopathies include targeted and generic methods as described in Section 3.5. However, due to the nature of the samples, reliability of the results should be ensured through the exclusion of maternal DNA contamination. To this end, laboratories analyse polymorphic genetic loci such as short tandem repeats (STRs) or variable number tandem repeats (VNTRs), to differentiate foetal from maternal DNA [85,86]. However, some laboratories report methods that attempt to purify the CVS specimens through dissection to minimise contamination, but it is not reliable and unsuitable when analysing amniotic fluid samples.

An essential component of applying PND is the acceptance of the potential termination of pregnancies. This varies globally, reflecting cultural, ethical and religious diversity [80]. While most pregnancies diagnosed with severe haemoglobinopathies tend to be terminated in many settings [83,87], others are not due to personal or religious beliefs. Of note is that in some Islamic communities, such as in Iran, termination for severe foetal genetic disorders, including transfusion-dependent thalassaemia, are permitted under specific religious rulings (fatwas) [85].

## 4. Discussion

Haemoglobinopathies were historically concentrated in the so-called “malaria belt of the world”, but recent migration and population movement have changed their global epidemiological distribution and demographics, prompting the WHO to recognise these disorders as an emerging global health concern. Prevention remains a cornerstone strategy, aiming to reduce affected births, support informed reproductive decisions, and ultimately improve patient outcomes. In this changing landscape, harmonising screening and diagnostic guidelines for haemoglobinopathies has become increasingly important. This literature review, conducted under the HELIOS COST Action WG1, highlights the substantial variability in current diagnostic practices and prevention strategies for haemoglobinopathies across countries, reflecting differences in healthcare infrastructure, local disease prevalence, resource availability, and socio-economic and cultural factors.

While many high-income and historically endemic countries have successfully implemented carrier screening and prevention strategies for up to four decades, the review highlights that many LMICs continue to face substantial barriers that hinder the establishment of standardised universal diagnostic laboratory methods and prevention programmes. Such barriers include underdeveloped disease-control policies, limited access to diagnostic tools due to restricted laboratory infrastructure [39,88], shortages of suitably trained professionals, gaps in awareness among general health providers and also low public awareness [89]. Additional challenges include high consanguinity rates in some populations [90] and ongoing global migration from high-prevalence regions to areas unprepared for haemoglobinopathy care [40,91]. Finally, besides cultural, religious, and socio-economic factors, the uptake and overall success of carrier testing and prevention is influenced by the strategic design and accessibility, including whether testing is voluntary or mandatory, or national versus regional [92].

Despite so many disparities, tiered screening strategies remain the most practical and cost-effective approach, particularly in resource-limited settings. These typically begin with basic haematologic testing (CBC, MCV, MCH, and haemoglobin typing), followed by molecular testing when indicated. These adaptable approaches have been successfully incorporated into premarital, school-based, and antenatal carrier screening frameworks, demonstrating a measurable impact on reducing the incidence of thalassaemia [93,94]. Migration and demographic changes may further complicate screening strategies by broadening mutation spectrums, making generic, rather than targeted, molecular approaches increasingly relevant. Overall, the choice of molecular testing protocols depends on population-specific mutation diversity, regional screening practices, and available resources. In many developing nations, targeted panels remain the mainstay due to affordability and the focus on prevalent local variants, while high-throughput and broad methods, such as next-generation or long-read sequencing, can enhance diagnostic capabilities and precision, and additionally detect rare or complex genotypes. It is apparent, however, that the adoption of NGS and/or LRS methods is limited by cost, infrastructure, and technical capacity, particularly in LMICs. Overall, tiered strategies combining haematologic pre-screening with targeted molecular testing continue to serve as the cornerstone of most current preventive programmes.

Epidemiological studies and well-curated variant registries are essential to support population-specific diagnostic protocols and cross-border harmonisation. Fragmented national data limit the understanding of mutation spectra, whereas linking local registries to global platforms such as HbVar, ITHANET, ClinVar, and the Global Globin Network (GGN) can help towards improving data standardisation, interoperability and quality. The GGN, operating under the Human Variome Project, promotes the use of existing infrastructures like the Leiden Open Variation Database (LOVD) to facilitate data sharing, particularly in LMICs [95]. Quality assurance, including reproducible HbA_2_ measurements, validated workflows, and expert-guided variant interpretation [96], is critical to ensure consistency across laboratories and support evidence-based policy and clinical decision-making [37].

In the light of so many challenges underscored in this review, harmonising international efforts for haemoglobinopathy prevention and care, although a necessity, presents a formidable task. While haemoglobinopathies are globally distributed, certain variants remain concentrated in specific ethnic groups [87], although demographic shifts, migration, and intermarriage have broadened the spectrum of haemoglobinopathies in many geographical regions, complicating the maintenance of tailored screening and diagnostic protocols.

Enhancing diagnostic capabilities, expanding access to carrier screening, raising public awareness, and implementing guidelines that combine harmonised principles with local adaptability are also essential components toward equitable care. While advanced technologies such as NGS and LRS promise unprecedented diagnostic precision, their routine adoption must be balanced with resource constraints, clinical utility, and ethical considerations.

In conclusion, for the immediate future, effective prevention will continue to rely on tiered strategies that integrate haematological pre-screening thresholds, molecular genotyping, prenatal diagnosis, and comprehensive epidemiological insights. The authors propose a two-pronged approach towards more harmonised guidelines: (1) core guidelines, outlining essential principles, minimum standards, and workflows that apply broadly across regions and form the backbone of good practice within health systems, and (2) context-adapted guidelines that allow protocols to be tailored to the capacities of individual laboratories, available instrumentation, and local disease prevalence, ensuring that recommendations are practical and implementable in diverse settings. Regardless of the prevention policy model adopted, the fundamental aim of these preventive strategies remains the same: to contribute towards the optimisation of clinical management through reducing the incidence of thalassaemias and other haemoglobinopathies by the timely identification of at-risk carrier-couples, supported with thorough counselling for informed reproductive choices, including prenatal diagnosis, or preimplantation genetic testing.

## 5. Conclusions and Future Directions

This review outlines current prevention frameworks for haemoglobinopathies and examines some emerging technologies that have the potential to transform both screening and diagnosis. Such emerging approaches include short and long next-generation sequencing (NGS or LRS), which were covered in the review, and also newborn screening (NBS) and noninvasive prenatal testing (NIPT). However, NBS and NIPT were not addressed in this review as their integration into routine diagnostic practice remains controversial and not yet widely implemented, but we considered that their inclusion in an expert comment was justified.

- **NGS-based methods:** NGS-based methods for identifying SNVs and SVs in the globin gene clusters include the more established short-read NGS protocols and the emerging LRS protocols. The capability to detect any variant within the genome, or within a specific genomic region of interest, in a single assay represents a great advantage compared to the traditional PCR-based genotyping methods. However, many of the variants detected are likely clinically silent or of uncertain significance, introducing a risk of overdiagnosis, associated with patient anxiety, unnecessary follow-up investigations, and an inefficient use of healthcare resources. Therefore, the applications of NGS/LRS require the concomitant use of stringent variant interpretation frameworks, continual reclassification efforts and refinement of variant databases to improve diagnostic accuracy over time. Furthermore, the current cost of NGS/LRS protocols limits their widespread adoption, particularly for population-level screening. While these methods generally enhance the diagnostic yield, their incremental benefits must be carefully weighed against higher costs, interpretative challenges, and the limited clinical relevance of many of the additional findings.
- **Newborn Screening (NBS)**: In many health systems, NBS has been widely adopted for the early detection of haemoglobinopathy patients. The use of NBS as an indirect means of screening for carrier parents remains debated in some countries (e.g., regarding who is informed and how), although it has been adopted in others (e.g., the UK, the Netherlands and some regions of Spain and Canada). Nevertheless, NBS should not be regarded as the primary strategy for haemoglobinopathy prevention. By design, it identifies affected newborns only after birth, offering a limited opportunity for informed reproductive decision-making and carrier counselling. Generally, NBS as an indirect means of carrier identification may be more appropriate and cost-effective in non-endemic countries, whereas the high(er) prevalence of carriers in endemic countries justifies a national (or regional) policy of directly screening for carriers.
- **Noninvasive Screening Method (cell-free foetal DNA (cffDNA)):** Once identified, a carrier couple may select the option of prenatal diagnosis, which traditionally includes an invasive biopsy of foetal genetic material (CVS or amniocentesis) followed by genetic analysis. The possibility of assessing cffDNA in maternal circulation is being adopted by some laboratories. Analysis of cffDNA, based on NGS technologies, was originally developed as a noninvasive screening method during pregnancy to detect genetic anomalies in the foetus, most notably aneuploidies [97]. However, there is a trend to extend the resolution of the NGS method and the analysis of the data to detect monogenic conditions alongside noninvasive aneuploidy screening. Of note is that screening is distinct from diagnosis, and a prerequisite for any technique to be characterised as a diagnostic tool requires studies to support evidence of validity (which to date are insufficient) and follow-up by a diagnostic test on an invasively acquired foetal sample (e.g., CVS or amniotic fluid) for all positive screening results. As with any reproductive genetic test, couples should be fully counselled about the pros, cons and limitations of both noninvasive and invasive prenatal testing, although currently there are no best-practice recommendations for the former.

In summary, the widespread implementation of new technology-driven applications should not be regarded as inevitable or universally advantageous. Careful evaluation of their technical robustness, interpretive complexity, resource implications, and ethical considerations is essential before integration into routine clinical practice.

## Figures and Tables

**Table 2:** Overview of Thalassaemia Diagnostic Methodologies Across Countries.

Country	Primary Methods Used For Presumptive Diagnosis	DNA Diagnosis	Reference(s)
**High-Income Countries**			
The Netherlands	CBC, Hb typing (HPLC, CE)	Alpha-targeted panel (Gap-PCR, alpha triplication) followed by gene scanning (MLPA and α-sequencing)	[1]
England	CBC, Hb typing (HPLC, CE) and FOQ (HP countries) LP countries; FOQ and HP protocol if high risk	Sanger sequencing, Gap-PCR, alpha triplication	[9,51]
Cyprus	CBC, Hb typing (HPLC)	NGS (DEVYSER), Gap-PCR	Personal communication
Italy	CBC, Hb typing (HPLC, CE)	Targeted panel: RDB, Gap-PCR Gene scanning: MLPA, Sanger sequencing, NGS	[11]
South Korea	CBC, Hb typing (HPLC)	Targeted panel: RDB, ARMS-PCR, Gap-PCR, Southern blotting Gene scanning: MLPA, Sanger sequencing, NGS	[52]
**Upper-Middle-Income Countries**			
Malaysia	CBC, Hb typing (HPLC, CE)	RDB- beta thalassaemia followed by gene scanning (β-sequencing, MLPA and DEVYSER) Alpha- Targeted panel (Gap-PCR) followed by gene scanning (MLPA and α-sequencing)	[29,53]
China	CBC, Hb typing (CE)	NGS (Combined NGS and Gap-PCR), RDB, HRM, Sanger sequencing MLPA, CATSA	[54,55,56]
Thailand	CBC, Hb typing, DCIP	Targeted panel: HRM, RDB, Gap-PCR, ARMS-PCR Gene scanning: MLPA, Sanger sequencing	[57]
**Lower-Middle-Income Countries**			
Indonesia	CBC, Hb typing (HPLC, CE, electrophoresis)	Sanger sequencing Not covered by National Health Insurance and high cost Mentzner Index (MI) and Shine & Lal Index are utilised to differentiate β-thalassaemia and iron deficiency anaemia	[58,59,60]
Morocco	CBC, Hb typing (CE)	Sanger sequencing, Gap-PCR	[61]
India	CBC, Hb typing (HPLC, CE)	Gap-PCR, ARMS-PCR, RDB, Sanger sequencing	[62]

**Table 3:** Comparison between Molecular Screening Methodologies for Haemoglobinopathies.

Method	Advantages	Limitations	Best Use Case (with References)
**Detection of Single Nucleotide Variations (SNVs)**			
ARMS-PCR	Precise for known SNVs Affordable and widely used Simple, rapid and inexpensive	Limited to known point mutations Requires prior mutation knowledge	β-thalassaemia carrier detection [6,44,45]
Reverse Dot Blot (RDB)	Multiplex detection Inexpensive Simple, rapid and reliable Automation workflow	Requires infrastructure and expertise Cannot detect novel variants	Screening in genetically diverse populations [41,44,52]
Sanger Sequencing	Gold standard for mutation confirmation Detects rare/novel mutations	Expensive Labour-intensive (particularly data interpretation) Not ideal for high throughput	Confirming ambiguous results and rare variants [1,6]
**Detection of Copy Number Variations (CNVs)**			
Gap-PCR	High specificity for known deletions Low cost Rapid results Can be designed as multiplex	Amplification of GC-rich region technically difficult Limited for deletions with known breakpoint sequences	Population screening for common α-thalassaemia deletions [7,41]
MLPA	High multiplex capacity Detects duplications	Costly for fragment analysis Break-point ambiguity Probe sensitivity to mismatches Stringent sample quality Unable to detect balanced rearrangement	Detection of atypical CNVs or in unresolved cases [63]
**Detection of SNVs and CNVs**			
High-resolution melting analysis (HRM)	Ability to detect SNVs and CNVs in one platform Rapid (does not require any post-PCR analysis)	Technically demanding Requires specialised instrument	[33,57,64]

**Table 4:** Comparison of emerging technologies for molecular diagnosis of haemoglobinopathies.

Technology	Principle	Main Applications	Advantages	Limits	Current Status/ Clinical Availability
Targeted NGS	Massive sequencing of target genes	Simultaneous identification of variants in *HBA1*, *HBA2*, *HBB*, *HBD*, *HBG1*, and *HBG2* genes and regulatory regions	Fast, moderate cost, high throughput; detects common/rare variants and complex thalassaemia with high precision	Limited in resolving repetitive regions (e.g., Alu/SINE in *HBA1*/*HBA2*), affecting α-thalassaemia analysis	Widely used in clinical settings [28,54,71,72]
TGS-PacBio (HiFi)	High-fidelity long reads	Rare variants, complex rearrangements, complete α/β haplotypes	Long, highly accurate reads (>99.9%); resolves repeats and complex variants; excellent for haplotype phasing	Higher cost; requires specialised equipment and bioinformatics expertise	Expanding use, mainly in research [56]
TGS-Oxford Nanopore (ONT)	Ultra-long nanopore reads	Rare variants, complex rearrangements, complete α/β haplotypes	Ultra-long reads; portable; real-time sequencing; detects epigenetic modifications directly	Higher raw error rate; requires extensive error correction and bioinformatics skills	Expanding use, mainly in research [73,74]
CATSA	RNA capture and reverse transcription with switching primer	Expression analysis detecting functional variants from mature red blood cell RNA in *HBA*/*HBB* genes	Detects all thalassaemia variant types (SNVs, indels, CNVs, and SVs) with high accuracy using multiplex PCR and TGS	Complex library preparation and bioinformatics analysis	Emerging, limited clinical adoption [27,69,75]
WES	Sequencing of all exons (coding regions)	Detection of mutations in globin and modifier genes	Lower cost than WGS; good coverage of coding regions	Omits intronic/regulatory regions and misses large deletions, limiting use in thalassaemia diagnosis	Useful as a first line in clinical genetics [76,77]
WGS	Whole genome sequencing	Unresolved/complex cases: detects non-coding variants, modifiers, and CNVs	Comprehensive screening (SNPs, indels, CNVs, SVs); useful in identifying modifiers	High cost; large volume of data; complex analysis and interpretation	High utility in research and difficult cases [78]

NGS: Next-Generation Sequencing; TGS: Third-Generation Sequencing; HiFi: High-Fidelity; ONT: Oxford Nanopore Technologies; CATSA: Comprehensive Analysis of Thalassemia Alleles; WES: Whole Exome Sequencing; WGS: Whole Genome Sequencing; SNVs: Single Nucleotide Variants; Indels: Insertions and Deletions; CNVs: Copy Number Variations; SVs: Structural Variants; α/β haplotypes: Alpha/Beta globin gene haplotypes; and Alu/SINE: Types of repetitive DNA elements (Alu and Short Interspersed Nuclear Elements).

## Data Availability

No new data were created or analyzed in this study. Data sharing is not applicable to this article.

## Supplementary Materials

The following supporting information can be downloaded at: https://www.mdpi.com/article/10.3390/ijms27093916/s1. References [22,81,98,99,100,101,102,103,104,105,106,107,108,109,110,111,112,113,114,115,116,117,118,119,120,121,122,123,124,125,126,127,128,129,130,131,132,133,134,135,136,137,138,139,140,141] are cited in the Supplementary Material.
