# Supplementary material for: Mapping Current and Emerging Laboratory Techniques for Haemoglobinopathy Carrier Detection and Prevention: A Narrative Review from the HELIOS Action
Source: Int J Mol Sci. 2026 Apr 28;27(9):3916. doi: 10.3390/ijms27093916 (PMC13164388; doi:10.3390/ijms27093916)
Supplement: Supplementary file 1 [file ijms-27-03916-s001.zip › ijms-4213960-supplementary.pdf]

Supplementary Data:

Supplementary Table S1: Thalassaemia carriers as reported in haemoglobinopathies prevention strategies related publications (2014–2025).

Publication Title	Study Period	Country	Cohort #	α-thal carriers	β-thal carriers	Total thalassaemia carriers reported	Thalassaemia Prevalence (%)
Nationwide Carrier Detection and Molecular Characterization of Beta-Thalassemia and Hemoglobin E Variants in Bangladeshi Population[98]	2018-2020	Bangladesh	1877	2	223	225	12
Thalassemia in Asia 2021 Thalassemia in Brunei Darussalam[99]	2009-2017	Brunei	8266	755	665	1420	17
Back-to-Back Comparison of Third-Generation Sequencing and Next-Generation Sequencing in Carrier Screening Thalassemia[90]	2020	China	1122	575	173	748	67
Combined Use of Gap-PCR and Next-Generation Sequencing Improves Thalassaemia Carrier Screening Among Premarital Results in China[40]	2017-2018	China	944	207	70	277	29
Evaluation of Intervention Strategy of Thalassemia for Couples of Childbearing Ages in Centre of Southern China[91]	2016-2019	China	3022	233	91	324	11

Expanded Carrier Screening Using Next-Generation Sequencing of 123 Hong Kong Chinese Families: A Pilot Study[88]	2016-2017	China	143	22	10	32	22
Gene Mutation Spectrum of Thalassemia Among Children in Yunnan Province[39]	2014-2018	China	3539	1544	1910	3454	98
Genotype-Phenotype Correlation Analysis of Patients with Thalassemia in Quanzhou City, Southeast of China[89]	2019-2022	China	2997	668	345	1013	34
Invasive Molecular Prenatal Diagnosis of Alpha and Beta Thalassemia Among Hakka Pregnant Women[100]	2014-2017	China	467	184	44	51	11
Molecular Characterization of Hemoglobinopathies and Thalassemias in Northern Guangdong Province, China[18]	2018-2020	China	10285	1340	2162	652	6
Molecular Prevalence of HBB-Associated HemoglobiNopathy Among Reproductive-Age Adults and the Prenatal DiagNosis in Jiangxi Province, Southern Central China[101]	2015-2021	China	136149	125	2358	2483	2

Molecular Spectrum and Prevalence of Thalassemia Investigated by Third-Generation Sequencing in the Dongguan Region of Guangdong Province, Southern China[102]	2020-2022	China	2716	153	105	257	9
Molecular Spectrum, Ethnic and Geographical Distribution of Thalassemia in the Southern Area of Hainan, China[48]	2019-2021	China	9813	5812	369	6181	63
Mutation Spectrum and Erythrocyte Indices Characterisation of α-Thalassaemia and β-Thalassaemia in Sichuan Women in China: A Thalassaemia Screening Survey 42 155 Women[55]	2017-2019	China	42155	29382	12056	41438	98
Next-Generation Sequencing Analysis of the Molecular Spectrum of Thalassemia in Souther Xiangxi, China[107]	2019-2021	China	136312	14298	4921	19219	14
Next-Generation Sequencing Improves Molecular Epidemiological Characterization of Thalassemia in Chenzhou Region, P.R. China[103]	2015-2017	China	15807	943	708	1651	10
Screening for Thalassemia Carriers Among the Han Population of Childbearing Age in Southwestern of China[104]	2022-2023	China	1093	84	43	127	12

Screening of Some Indicators for Alpha-Thalassemia in Fujian Province Of Southern China[105]	2016-2019	China	13294	2658	:	2658	20
Beta Thalassemia Carrier Rate: Problem Burden Among High School Children[106]	2016-2020	Egypt	4320	:	265	265	6
Clinical to Molecular Screening Paradigm for Beta-Thalassemia Carriers[22]	2009-2010	Egypt	1627	36	63	99	6
Screening for B-Thalassemia Carrier Among Students in a Secondary School In Diarb Negm, Sharkia [107]	2016-2017	Egypt	614	:	52	52	9
A Comprehensive Screening Program for β-Thalassemia and Other Hemoglobinopathies in the Hooghly District of West Bengal, India, Dealing with 21 137 Cases[108]	2012-2015	India	21137	:	1968	1968	9
Incidence af β-Thalassemia Carriers in Muzaffaraba Azad Kashmir[109]	:	India	500	:	28	28	6
Population Screening and Prevention Strategies for Thalassemias and Other Hemoglobinopathies of Eastern India: Experience 18,166 Cases[110]	1999-2011	India	18166	:	2092	2092	12

Prevalence of β-Haemoglobinopathies in Eastern India and Development of A Novel Formula for Carrier Detection[111]	2014-2019	India	21695	:	2170	2170	10
Significance of Borderline Hba2 Levels in β-Thalassemia Carrier Screening[112]	2009-2013	India	205	:	168	168	82
Effectiveness of β-Thalassemia Prenatal Diagnosis in Southern Iran: Cohort Study[85]	2004-2012	Iran	1016	:	499	499	49
Prevalence of SS-Thalassemia Mutations Among Northeastern Iranian Population and Their Impacts on Hematological Indices and Application of Prenatal Diagnosis, A Seven-Years Study[113]	2011-2018	Iran	1593	:	1273	1273	80
Premarital Hemoglobinopathy Screening Program Results of A Province in the Black Sea Region of Turkey: Three Year Experience[114]	2019-2021	Iraq	52338	:	696	696	1
Prevalence and Molecular Characterization of Beta-Thalassemia in Kirkuk Province of Northern Iraq[115]	2022-2023	Iraq	3954	:	119	119	3
Beta-Hemoglobinopathies in the Lao People's Democratic Republic: Molecular Diagnostics and Implication for a Prevention and Control Program[116]	2002-2003	Laos	519	:	233	233	45

Current Status of Thalassemia in Lao People's Democratic Republic[117]	2021-2022	Laos	307	33	49	82	27
Thalassemia Distribution Based on Screening Programs in the Population of the East Malaysian State of Sabah[118]	2013	Malaysia	645	:	151	151	23
Genetic Epidemiology of β-Thalassemia in the Maldives: 23 Years of A β-Thalassemia Screening Program[119]	1992-2015	Maldives	110504	:	17902	17902	16
Alpha-Thalassemia ιn North Morocco: Prevalence αnd Molecular Spectrum[120]	2015-2016	Morocco	1658	16	:	16	1
Premarital Genetic Screening for β-Thalassemia Carrier Status of Indexed Families Using HbA2 Electrophoresis[94]	2011	Pakistan	98	:	57	57	58
Prevalence of Hemoglobinopathies (β-Thalassemia and Sickle Cell Trait) in the Adult Population of Al Majma'a[121]	2016-2019	Saudi Arabia	3755	:	38	38	1
Epidemiology of Thalassemia Among the Hill Tribe Population in Thailand[122]	:	Thailand	1200	62	55	117	10

Reliability of Hemoglobin A2 Value as Measured by the Premier Resolution System for Screening of β-Thalassemia Carriers[67]	2022	Thailand	418	:	133	133	32
The Shortcut Strategy for β-Thalassimia Prevention[57]	2015-2016	Thailand	1115	:	66	66	6
Prevalence of Hemoglobinopathies in Premarital Screening in the Province of Nigde, Turkey[123]	2019-2021	Turkey	2013	:	53	53	3
Thalassemias and Hemoglobinopathies in Turkey[124]	2003-2013	Turkey	380000	:	16340	16340	4

Supplementary Figure S1. Country-wise distribution of molecular haemoglobin β-globin gene variants reported in haemoglobinopathies prevention strategies related publications (2014-2025). (Note: traditional variant nomenclature has been used)

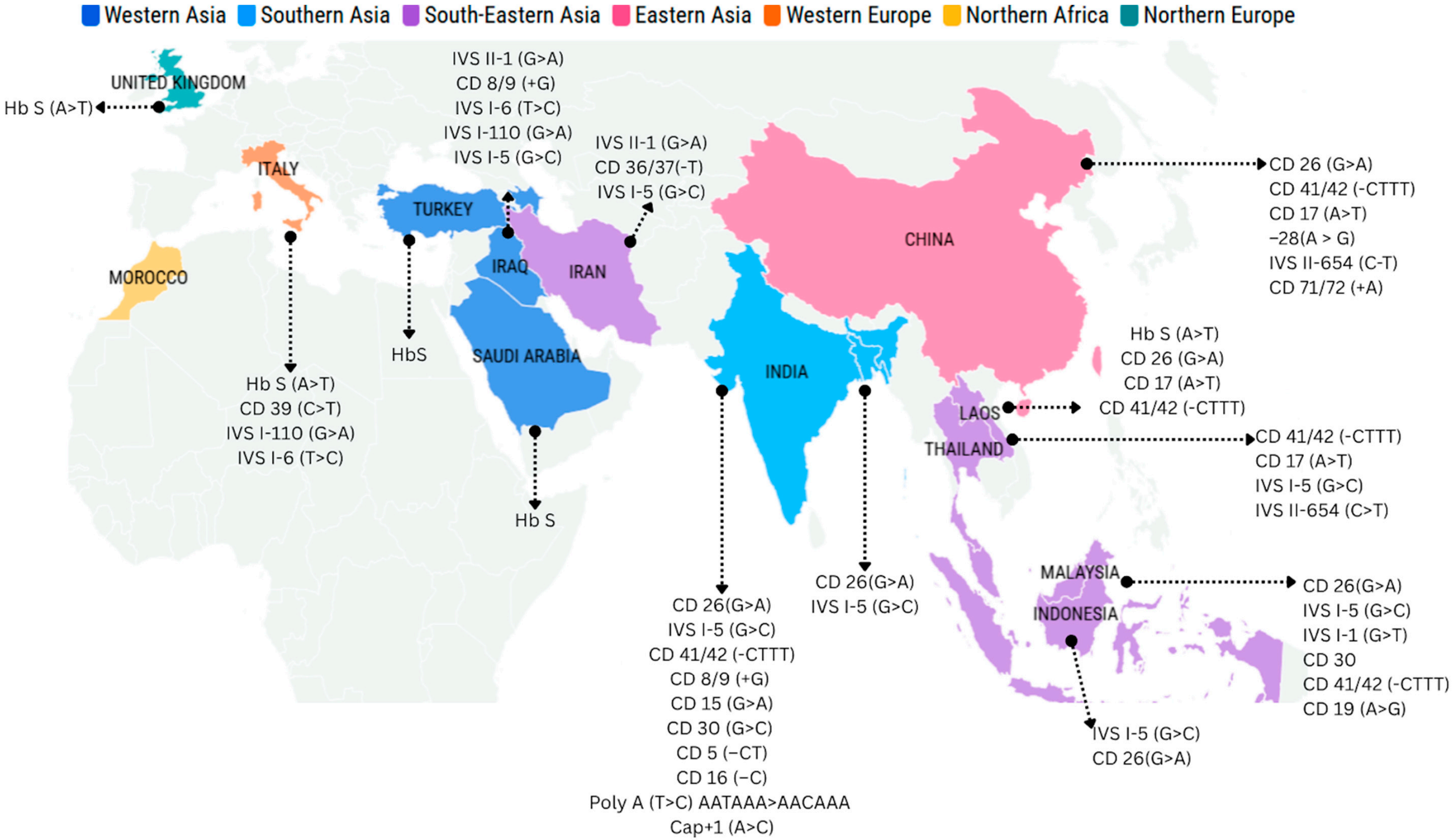

Supplementary Figure S2. Country-wise distribution of molecular haemoglobin α-globin gene variants reported in haemoglobinopathies prevention strategies related publications (2014-2025). (Note: traditional variant Nomenclature has been used)

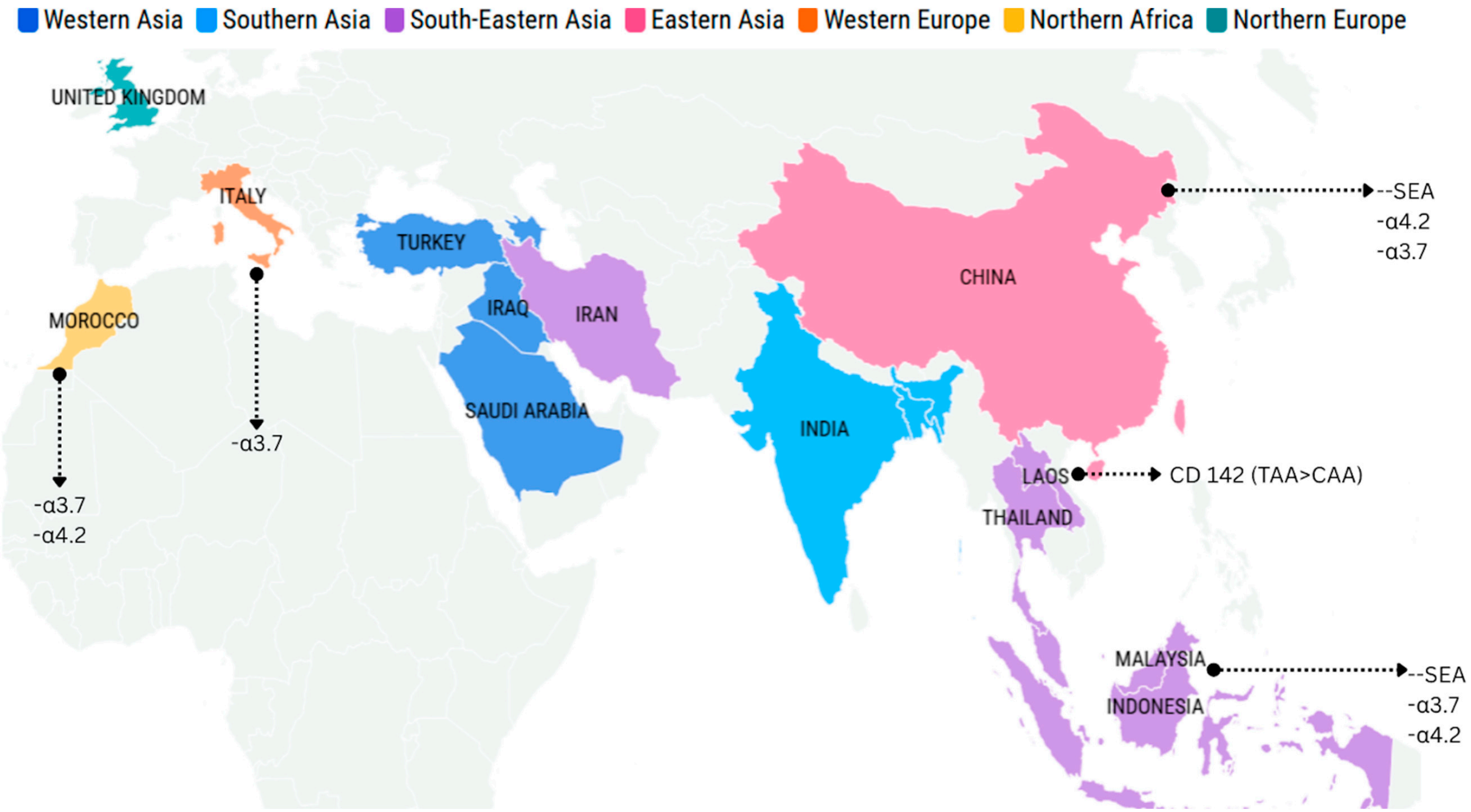

Supplementary Figures S1 & S2: Global distribution of globin gene variants from 2014–2025 prevention strategies related publications

From the curated publications on prevention strategies spanning 2014–2025, we gathered data on the prevalence of both β- and α-globin gene variants (Figure S1 and Figure S2, respectively). As illustrated in the figures, the β-globin gene exhibits broad molecular diversity across regions, whereas α-globin gene variants appear more consistent. The most common α-globin deletions, -α3.7kb or -α4.2kb, remove a single α-globin gene and are prevalent in most areas. The analysis also highlights that the β-globin variants are more extensively diagnosed and reported, while relatively few studies focus on α-globin variants.

Supplementary Table S2: Molecular variants per country/region as reported in haemoglobinopathies prevention strategies related publications (2014–2025).

IthaID indicates the corresponding entry in the ITHANET Portal [https://www.ithanet.eu/db/ithagenes?action=list].

* denotes an unspecified nucleotide change.

Publication Title	Country	α-thal variants	β-thal variants
Update on Prevention of Hemoglobinopathies in Azerbaijan[125]	Azerbaijan	:	CD 8 (-AA) (IthaID:61)<br>IVS II-1 (G>A) (IthaID:200)<br>IVS I-110 (G>A) (IthaID:113)
Nationwide Carrier Detection and Molecular Characterization of β-Thalassemia and Hemoglobin E Variants in Bangladeshi Population[98]	Bangladesh	:	CD 26 (G>A) (IthaID:88)<br>IVS I-5 (G>C) (IthaID:107)
Thalassemia in Asia 2021 Thalassemia in Brunei Darussalam[99]	Brunei	:	CD 26 (G>A) (IthaID:88)
Back-To-Back Comparison of Third-Generation Sequencing and Next-Generation Sequencing in Carrier Screening of Thalassemia[90]	China	-α4.2 (IthaID:301)<br>-α3.7	:
Combined Use of Gap-PCR and Next-Generation Sequencing Improves Thalassaemia Carrier Screening Among Premarital Adults in China[40]	China	--SEA (IthaID:309)<br>-α3.7	:

Gene Mutation Spectrum of Thalassemia Among Children in Yunnan Province[39]	China	--SEA (IthaID:309)<br>-α3.7	CD 17 (A>T) (IthaID:77)<br>CD 41/42 (-CTTT) (IthaID:147)<br>IVS II-654 (C>T) (IthaID:211)
Genotype-Phenotype Correlation Analysis of Patients With Thalassemia In Quanzhou City, Southeast of China[89]	China	--SEA (IthaID:309)<br>-α3.7	CD 41/42 (-CTTT) (IthaID:147)<br>IVS II-654 (C>T) (IthaID:211)
Invasive Molecular Prenatal Diagnosis of α- and β-Thalassemia among Hakka Pregnant Women[100]	China	--SEA (IthaID:309)	:
Molecular Characterization of Hemoglobinopathies and Thalassemias in Northern Guangdong Province, China[18]	China	–SEA (IthaID:309)<br>-α3.7<br>-α4.2 (IthaID:301)	CD 17 (A>T) (IthaID:77)<br>CD 41/42 (-CTTT) (IthaID:147)<br>IVS II-654 (C>T) (IthaID:211)<br>-28 (A>G) (IthaID:29)
Molecular Prevalence of HBB-Associated Hemoglobinopathy among Reproductive-Age Adults and the Prenatal Diagnosis in Jiangxi Province, Southern Central China[101]	China	:	CD 17 (A>T) (IthaID:77)<br>CD 41/42 (-CTTT) (IthaID:147)<br>IVS II-654 (C>T) (IthaID:211)

Molecular Spectrum and Prevalence of Thalassemia Investigated by Third-Generation Sequencing in the Dongguan Region of Guangdong Province, Southern China[102]	China	--SEA (IthaID:309)<br>-α3.7	CD 41/42 (-CTTT) (IthaID:147)<br>IVS II-654 (C>T) (IthaID:211)
Molecular Spectrum, Ethnic and Geographical Distribution of Thalassemia in the Southern Area of Hainan, China[48]	China	-α4.2 (IthaID:301)<br>-α3.7	CD 17 (A>T) (IthaID:77)<br>CD 41/42 (-CTTT) (IthaID:147)<br>IVS II-654 (C>T) (IthaID:211)<br>-28 (A>G) (IthaID:29)<br>CD 71/72 (+A) (IthaID:177)<br>CD 26 (G>A) (IthaID:88)
Mutation Spectrum and Erythrocyte Indices Characterisation af α-Thalassaemia and β-Thalassaemia in Sichuan Women in China: A Thalassaemia Screening Survey 42 155 Women[55]	China	-α3.7<br>--SEA (IthaID:309)	CD 17 (A>T) (IthaID:77)<br>IVS II-654 (C>T) (IthaID:211)<br>CD 41/42 (-CTTT) (IthaID:147)
Next-Generation Sequencing Analysis of the Molecular Spectrum of Thalassemia in Southern Jiangxi, China[103]	China	--SEA (IthaID:309)<br>-α3.7	IVS II-654 (C>T) (IthaID:211)
Screening for Thalassemia Carriers Among the Han Population of Childbearing Age in Southwestern of China[26]	China	--SEA (IthaID:309)	CD 17 (A>T) (IthaID:77)

		-α3.7	CD 41/42 (-CTTT) (IthaID:147)
Screening of Some Indicators for α-Thalassemia in Fujian Province of Southern China[105]	China	--SEA (IthaID:309)<br>-α3.7	:
A Comprehensive Screening Program for β-Thalassemia and Other Hemoglobinopathies in the Hooghly District of West Bengal, India, Dealing with 21,137 Cases[108]	India	:	CD 26 (G>A) (IthaID:88)
Current Status of β-Thalassemic Burden in India[128]	India	:	IVS I-5 (G>C) (IthaID:107)<br>CD 41/42 (-CTTT) (IthaID:147)<br>CD 8/9 (+G) (IthaID:62)<br>CD 15 (G>A) (IthaID:72)<br>CD 30 (G>C)*<br>CD 5 (-CT) (IthaID:54)<br>CD 16 (-C) (IthaID:75)
Significance of Borderline HbA2 Levels in β-Thalassemia Carrier Screening[112]	India	:	IVS I-5 (G>C) (IthaID:107)<br>Poly A (T>C) AATAAA>AACAAA (IthaID:272)

			CAP+1 (A>C) (IthaID:34)
Molecular Scanning of beta-Thalassemia in the Southern Region of Central Java, Indonesia; a Step Towards a Local Prevention Program[130]	Indonesia	:	IVS I-5 (G>C) (IthaID:107)<br>CD 26 (G>A) (IthaID:88)
Effectiveness of β-Thalassemia Prenatal Diagnosis in Southern Iran: Cohort Study [85]	Iran	:	IVS II-I (G>A) (IthaID:200)<br>CD 36/37 (-T) (IthaID:134)<br>IVS I-5 (G>C) (IthaID:107)
Prevalence Of B-Thalassemia Mutations among Northeastern Iranian Population and Their Impacts on Hematological Indices and Application of Prenatal Diagnosis, A Seven-Years Study[113]	Iran	:	IVS II-1 (G>A) (IthaID:200)<br>IVS I-5 (G>C) (IthaID:107)
Prevalence and Molecular Characterization of β-Thalassemia in Kirkuk Province of Northern Iraq[115]	Iraq	:	IVS II-1 (G>A) (IthaID:200)<br>CD 8/9 (+G) (IthaID:62)<br>IVS I-6 (T > C) (IthaID:111)<br>IVS I-110 (G>A) (IthaID:113)<br>IVS I-5 (G>C) (IthaID:107)
Incidence of Haemoglobinopathies in Sicily: The Impact of Screening and Prenatal Diagnosis[126]	Italy	-α3.7	CD 6 (A>T) (IthaID:824)<br>CD 39 (C>T) (IthaID:142)<br>IVS I-110 (G>A) (IthaID:113)

			IVS I-6 (T>C) (IthaID:111)
β-Hemoglobinopathies in the Lao People's Democratic Republic: Molecular Diagnostics and Implication for A Prevention and Control Program[116]	Laos	:	CD 26 (G>A) (IthaID:88)<br>CD 17 (A>T) (IthaID:77)<br>CD 41/42 (-CTTT) (IthaID:147)
Current Status of Thalassemia in Lao People's Democratic Republic[117]	Laos	CD 142 (T>C) (IthaID:418)	CD 6 (A>T) (IthaID:824)<br>CD 26 (G>A) (IthaID:88)
Application of Targeted Next-Generation Sequencing for the Investigation of Thalassemia in a Developing Country: A Single Center Experience[29]	Malaysia	--SEA (IthaID:309)<br>-α3.7<br>-α4.2 (IthaID:301)	CD 26 (G>A) (IthaID:88)<br>IVS I-5 (G>C) (IthaID:107)<br>IVS I-1 (G>T) (IthaID:102)
Thalassemia in Malaysia[127]	Malaysia	:	CD 26 (G>A) (IthaID:88)<br>CD 19 (A>G)*<br>IVS I-1 (G>T) (IthaID:102)<br>IVS I-5 (G>C) (IthaID:107)<br>Poly A (A>G) (AATAAA>AATAGA) (IthaID:275)

Genetic Epidemiology of β-Thalassemia in the Maldives: 23 Years of A β-Thalassemia Screening Program[119]	Maldives	:	IVS I-5 (G>C) (IthaID:107)<br>IVS II-1 (G>A) (IthaID:200)<br>CD 30 (G>C)*<br>CD 41/42 (-CTTT) (IthaID:147)
Alpha-Thalassemia in North Morocco: Prevalence and Molecular Spectrum[120]	Morocco	-α3.7<br>-α4.2 (IthaID:301)	:
Prevalence of Hemoglobinopathies (β-Thalassemia and Sickle Cell Trait) in the Adult Population of Al Majma'ah, Saudi Arabia[121]	Saudi Arabia	:	CD 6 (A>T) (IthaID:824)
Reliability of Hemoglobin A2 Value as Measured by the Premier Resolution System for Screening of β-Thalassemia Carriers[25]	Thailand	:	CD 41/42 (-CTTT) (IthaID:147)<br>CD 17 (A>T) (IthaID:77)
Revisiting and Updating Molecular Epidemiology of α-thalassemia Mutations in Thailand Using MLPA and New Multiplex Gap-PCR for Nine α-thalassemia Deletion[33]	Thailand	--SEA (IthaID:309)	:
Thalassemia in Thailand[122]	Thailand	:	CD 41/42 (-CTTT) (IthaID:147)<br>CD 17 (A>T) (IthaID:77)<br>IVS I-5 (G>C) (IthaID:107)

			IVS II-654 (C>T) (IthaID:211)
The Shortcut Strategy for β-Thalassemia Prevention[57]	Thailand	:	CD 17 (A>T) (IthaID:77)<br>CD 41/42 (-CTTT) (IthaID:147)<br>IVS I-1 (G>T) (IthaID:102)
Thalassemias and Hemoglobinopathies in Turkey[124]	Turkey	:	CD 6 (A>T) (IthaID:824)
Sickle Cell Disease and Thalassaemia Antenatal Screening Programme in England over 10 Years: A Review from 2007/2008 to 2016/2017[129]	United Kingdom	:	CD 6 (A>T) (IthaID:824)

Supplementary Table S3: Search strategy. Time period: 2014-2024 (December)

No	Search	Search Syntax	Results
**1**	**PubMED**	**(laboratory AND (guideline OR policy)) AND (prevention OR diagnosis OR screening) AND (carrier OR carriers OR prenatal) AND (thalassemia OR thalassaemia OR "sickle cell" OR SCD OR hemoglobinopath* OR haemoglobinopath*)**	**37 results**
**2**	**PubMED**	**(guideline* OR "best practice guideline*" OR policy OR recommendation*) AND ((screening OR diagnosis) AND (carrier* OR premarital OR antenatal OR prenatal)) AND (sickle OR thalassemia OR thalassaemia OR hemoglobinopath* OR haemoglobinopath*)**	**349 results**
**3**	**PubMED**	**"HemoglobiNopathies"[MeSH] OR hemoglobiNopathy[Title/Abstract]) AND ("screening"[Title/Abstract] OR "prevention"[Title/Abstract]) AND ("guidelines"[Title/Abstract] OR "recommendations"[Title/Abstract]) AND ("2014/01/01"[PDAT] : "2025/12/31"[PDAT]**	**100 results**

**4**	**Medline**	**Thalassemia/ OR thalassaemia.*tw. OR beta -thalassemia/ OR alpha-thalassemia AND screening.tw. OR diagnosis.tw. AND prevention.tw.**	**464 results**
**5**	**Medline**	**Thalassemia/ OR thalassaemia.*tw. OR beta -thalassemia/ OR alpha-thalassemia AND screening.tw. OR prenatal diagnosis.tw. AND prevention.tw.**	**553 results**
**6**	**Medline**	**(sickle cell OR haemoglobinopath* OR thalassaemia OR beta-thalassaemia OR alpha-thalassaemia).tw. AND screening.tw. OR diagnosis.tw. OR (antenatal or prenatal or carrier* or premarital).tw AND prevention*.tw. OR best practice guideline.tw. OR guideline.tw.**	**498 results**
**7**	**Publish and Perish**	**(guideline* OR "best practice guideline*" OR policy OR recommendation*) AND ((screening OR diagnosis) AND (carrier* OR premarital OR antenatal OR prenatal)) AND (sickle OR thalassemia OR thalassaemia OR haemoglobinopath* OR haemoglobinopath*)**	**500 results**

Supplementary Table S4: Prevention programs across regions and income levels.

**References**	**Country**	**Active Programs**	**Discontinued Programs**	**Active NBS**	**Discontinued NBS**	**Preconceptional screening**	**Premarital**	**Carrier screening**	**Antenatal screening**	**PND**	**Antenatal screening or PND (unspecified)**	**Cohort/ Focus group**	**Screening Status**	**Application**	**Region**	**Income group**
[82]	Angola	No			Yes										Sub-Saharan Africa	LMIC

[82,84]	Australia	Yes				Yes					Yes		V	R	East Asia & Pacific	HIC
[125]	Azerbaijan						Yes [2014]	Yes [2005; state program implemented]		Yes [2014]		At Risk Couples	M	N	Europe & Central Asia	UMIC
[82,131]	Bahrain	Yes		Yes [2007]			Yes [2004; M]	Yes [1998; Secondary school pupils]	Yes [1992]	Yes [2005]				N	Middle East & North Africa	HIC
[84]	Bangladesh	No													South Asia	LMIC
[82,140]	Belgium [Brussels]	Yes		Yes [1994]					Yes			Universal		R	Europe & Central Asia	HIC
[82]	Benin [Cotonou]			Yes [1993, pilot, R]											Sub-Saharan Africa	LMIC

[82,84]	Brazil	No		Yes [2001, M, N]											Latin America & Caribbean	UMIC
[84]	Brunei	No		No											East Asia & Pacific	HIC
[82]	Burkina Faso [Ouagadougou]	No			Yes [R/pilot]										Sub-Saharan Africa	Low income
[84]	Cambodia	No		No											East Asia & Pacific	LMIC
[45]	Canada	Yes		Yes		Yes						Targeted [at risk couples]	V	N	North America	HIC
[45,137]	China	Yes		No		Yes		Yes	Yes				V	R	East Asia & Pacific	UMIC

[45]	HONG KONG	Yes							Yes [2000]	Yes [if needed]			V	N	East Asia & Pacific	HIC
[82]	Colombia	No			Yes [2000-2014, R]										Latin America & Caribbean	UMIC
[82]	Costa Rica	No		Yes [M, N]											Latin America & Caribbean	UMIC
[82]	Cuba	Yes							Yes [1983]				V	N	Latin America & Caribbean	UMIC
[82,133]	Cyprus	Yes		No			Yes [1973]		Yes	Yes [1977]			M	N	Europe & Central Asia	HIC
[82]	Dominican Republic	No		Yes [2014, V, N]											Latin America &	UMIC

															Caribbean	
[82,84]	DR Congo	No		Yes [pilot, R]											Sub-Saharan Africa	Low income
[84]	Egypt	Yes		No					Yes	Yes			V	N	Middle East & North Africa	LMIC
[82]	France	No	Yes [1978-1985, Marseille (secondary school pupils)]	Yes [2024, Universal, V, N]											Europe & Central Asia	HIC
[134]	Germany			Yes [2020, Universal, N]											Europe & Central Asia	HIC
[82]	Ghana	No		Yes [1993, expanded in											Sub-Saharan Africa	LMIC

				2021, Universal, V, N]												
[82,81]	Greece	Yes					Yes [1974]		Yes [1974]	Yes [1974]			V	N	Europe & Central Asia	HIC
[82]	India	Yes						Yes [2023]				Targeted (high risk)	V	N	South Asia	LMIC
[84,136]	Indonesia	Yes						Yes [2025]			Yes [V, R]	Children	M	N	East Asia & Pacific	UMIC
[82,84,131]	Iran	Yes					Yes [1997]			Yes			M	N	Middle East & North Africa	UMIC
[82,131]	Iraq	Yes					Yes [2008]			Yes			M	R	Middle East & North Africa	UMIC

[82]	Israel	Yes				Yes [1980]			Yes [1980]			Targeted (high risk)	V	N	Middle East & North Africa	HIC
[82,133]	Italy	Yes					Yes [1975, Sardinia]	Yes [1975, school, Latium]	Yes [1975, Sardinia]	Yes [1983, Sicily]			V	R	Europe & Central Asia	HIC
[82,131]	Jordan	Yes					Yes [2004]			Yes			M	N	Middle East & North Africa	LMIC
[84]	Laos	Yes		Yes				Yes			Yes		PILOT	R	East Asia & Pacific	LMIC
[82]	Lebanon	Yes					Yes [1994]	Yes [V, general population and high risk groups]					M	N	Middle East & North Africa	LMIC
[82]	Malaysia	Yes					Yes [2004]	Yes [school-based	Yes [women, 2004]	Yes (upon request)			V	N	East Asia & Pacific	UMIC

								(2016), cascade]								
[82]	Maldives	Yes					Yes [1992, M since 2012]			Yes [established in 2012]		Universal	M	N	South Asia	UMIC
[82,134]	Malta	Yes		Yes [N, Universal]					Yes [1991]				V	N	Middle East & North Africa	HIC
[61]	Morocco	No		No				No							Middle East & North Africa	LMIC
[84]	Myanmar (Burma)	No													East Asia & Pacific	LMIC
[84]	Nepal	No													South Asia	LMIC
[82,84]	Netherlands	Yes		Yes [2007]		Yes [2007]		Yes (1970s upon request)	Yes	No			V	N	Europe & Central Asia	HIC

[84]	Nigeria	Yes		Yes					Yes (women)			Universal		N	Sub-Saharan Africa	LMIC
[82]	Oman	Yes			Yes [2005-2007, pilot]		Yes [1999]						V	R	Middle East & North Africa	HIC
[84,137]	Pakistan	Yes					Yes			Yes			V	R	South Asia	LMIC
[82]	Palestine	Yes					Yes [2000]						M	R	Middle East & North Africa	LMIC
[82,84]	Philippines	No		Yes [2014, N]											East Asia & Pacific	LMIC
[82]	Portugal	Yes					Yes		Yes				V	R	Europe & Central Asia	HIC
[138]	Qatar	Yes					Yes [2009]						M	N	Middle East &	HIC

															North Africa	
[82]	Saudi Arabia	Yes					Yes [2004]						M	N	Middle East & North Africa	HIC
[82,84]	Singapore	Yes						Yes[cascade, 1980]	Yes [women, 1997]				V	N	East Asia & Pacific	HIC
[84]	South Africa	No													Sub-Saharan Africa	UMIC
[82]	Spain	Yes		Yes [2003]			Yes [on demand]			Yes [on demand]		Targeted (high risk/ethnic)		N	Europe & Central Asia	HIC
[82,84]	Sri Lanka	Yes					Yes [2006, over age 15)	Yes [cascade]	No	NO (service availble)			V	N	South Asia	LMIC

[82]	Taiwan	Yes							Yes [women, 1993]			Universal	V	N	East Asia & Pacific	HIC
[82]	Tanzania	No			Yes [pilot]										Sub-Saharan Africa	LMIC
[82]	Thailand	Yes							Yes [women, 1997]	Yes [1997]			V	N	East Asia & Pacific	UMIC
[82]	Tunisia	No		Yes [pilot, R]											Middle East & North Africa	LMIC
[82,84]	Turkey	Yes					Yes [2003]			Yes			V	R	Europe & Central Asia	UMIC
[82,131]	United Arab Emirates	Yes		Yes			Yes [2011]			Yes [2005, V]			M	N	Middle East & North Africa	HIC

[82,84]	United Kingdom	Yes		Yes [2004]					Yes [women, 2004]			Universal	V	N	Europe & Central Asia	HIC
[82,133]	United States	Yes		Yes [2005, M, N]			Yes	Yes				Targeted (high risk)	V	R	North America	HIC
[82]	Uruguay	No		Yes [pilot]											Latin America & Caribbean	HIC
[84]	Vietnam	Yes												R	East Asia & Pacific	LMIC
**Other resources (manually added beyond the bibliography scan)**																
[139]	Canada	Yes		Yes		Yes						Targeted [at risk couples]	V	N	North America	HIC
[140]	India	Yes						Yes [2023]				Targeted (high risk)	V	N	South Asia	LMIC

[136]	Indonesia	Yes						Yes [2025]			Yes [V, R]	Children	M	N	East Asia & Pacific	UMIC
[141]	Tunisia	No		Yes [pilot, R]											Middle East & North Africa	LMIC

NBS=Newborn Screening; PND=Prenatal Diagnosis; M=Mandatory; V=Voluntary; R=Regional; N=National; LMIC=Low Middle Income Countries; UMIC=Upper Middle Income Countries; HIC=High Income Countries
